# Terroir-driven phenolic chemotypes and antioxidant/antimicrobial activities of Beldia *(Cannabis sativa L.)* seed extracts

**DOI:** 10.1186/s42238-026-00468-y

**Published:** 2026-07-18

**Authors:** Oumayma Boussetta, Oussama Khibech, Abdessadek Essadek, Shehdeh Jodeh, Meryem Idrissi Yahyaoui, Abdeslam Asehraou, Chin-Hung Lai, Mohammed Ramdani

**Affiliations:** 1 Laboratory of Applied Chemistry and Environment, Faculty of Sciences, Mohammed First University, Bd. Med VI, B.P. 717, 60000 Oujda, Morocco; 2 Laboratory of Applied Chemistry and Environment (LCAE-ECOMP), Faculty of Sciences, Mohammed First University, 60000 Oujda, PB Morocco; 3 Laboratory of Bioresources, Biotechnology, Ethnopharmacology and Health, Faculty of Sciences, Mohammed First University, Bd. Med VI, B.P. 717, 60000 Oujda, Morocco; 4 Department of Chemistry, An-Najah National University, P.O. Box 7, Nablus, Palestine; 5 Department of Medical Applied Chemistry, Chung Shan Medical University, Taichung, 402 Taiwan

**Keywords:** *Cannabis sativa L*, Geographical influence, Aqueous seed extracts, HPLC–DAD, Antioxidant activity, Antimicrobial activity

## Abstract

Geographical variation in the phenolic composition and bioactivity of Moroccan Beldia (*Cannabis sativa L*.) seeds remains insufficiently characterized, limiting their rational valorization as bioactive resources. This study investigated aqueous extracts from defatted seeds collected in Ketama, Taounate, and Ouazzane using HPLC–DAD phenolic profiling, antioxidant and antimicrobial assays, and molecular docking. Data are reported as mean ± SD (*n* = 3), and regional differences were evaluated by one-way ANOVA followed by Tukey’s test when applicable. Clear regional discrimination was observed. Ouazzane gave the highest extraction yield (8.08 ± 0.24%), total phenolic content (16.13 ± 0.48 mg GAE/100 g DM), total flavonoid content (6.78 ± 0.20 mg QE/100 g DM), and total antioxidant capacity (565.80 ± 5.90 mg AAE/g), whereas Ketama showed the strongest DPPH scavenging activity (IC₅₀ = 10.25 ± 0.03 µg/mL). HPLC–DAD revealed distinct chemotypes dominated by 4-hydroxybenzoic acid (33.26–36.63%), with Taounate enriched in vanillic acid (35.00%) and caffeic acid (20.44%). Antimicrobial assays showed the best MIC values against *Micrococcus luteus* (0.50 ± 0.01 mg/mL) and *Geotrichum candidum* (0.12 ± 0.00 mg/mL). Docking highlighted flavonoid-type constituents as the most plausible active contributors, with catechin hydrate and rutin showing the strongest affinity toward CYP51 (-9.6 kcal/mol). Moroccan terroir strongly shapes the functional profile of Beldia seed extracts and supports region-guided sourcing for future bioactive applications.

## Introduction

Hemp seeds from *Cannabis sativa L*. are increasingly recognized as valuable matrices not only because of their lipid fraction but also because of the phenolic constituents retained in defatted meals and polar extracts. Recent reviews and analytical studies indicate that cannabis seed-derived phenolics include hydroxybenzoic acids, hydroxycinnamic acids, flavonoids, and specialized lignanamides, and that the recovered profile depends strongly on extraction conditions and solvent polarity (Benkirane et al. 2026). These observations support the exploration of defatted seed residues as an underused source of water-soluble bioactive compounds (Kalinowska et al. 2022; Allay et al. 2024).

Geographical origin is another important determinant of seed composition. Moroccan studies have shown that both variety and growing area shape the phenolic fingerprints of northern hemp seeds and influence the composition of cannabis achenes and seed oils (Benkirane et al. 2023). For local landraces such as Beldia, region-resolved characterization is therefore necessary to understand how terroir-associated variation may affect chemical quality and biological performance (Ouhtit et al. 2024).

From a functional perspective, phenolic acids can contribute to antibacterial effects through membrane perturbation and intracellular leakage (Borges et al. 2013). In antifungal pharmacology, clinically used azoles such as fluconazole and voriconazole inhibit sterol 14α-demethylase (CYP51/ERG11), an essential cytochrome P450 enzyme in ergosterol biosynthesis (Kaluzhskiy et al. 2021; Rosam et al. 2020). This target is therefore relevant for interpreting whether seed phenolics may plausibly contribute to the antifungal effects observed in vitro.

However, previous Moroccan studies on *Cannabis sativa* seed extracts have largely focused on whole seeds, seed oil, or hydroalcoholic extracts, whereas aqueous decoctions prepared from defatted Beldia seeds remain comparatively underexplored. In particular, the extent to which regional origin influences the phenolic signatures and bioactivities of these water-soluble fractions has not been clearly established.

Accordingly, the present study compared aqueous extracts prepared from defatted Beldia seeds collected from Ketama, Taounate, and Ouazzane. We characterized extraction yield, total phenolic and flavonoid contents, HPLC–DAD phenolic profiles, antioxidant capacity, and antibacterial and antifungal activities. Molecular docking against bacterial DNA gyrase subunit B (4URO) and fungal CYP51 (5JLC) was used as a complementary mechanistic tool to prioritize the most plausible phytochemical contributors. To avoid overinterpretation, the regional comparisons are discussed as geography-associated differences rather than direct causal effects of specific environmental variables.

## Materials and Methods

### Plant material

Hemp *(Cannabis sativa L.)* seeds from the Moroccan Beldia landrace were collected in July 2023 from three mountainous cultivation areas in northern Morocco: Ketama (~ 1500 m a.s.l.; 34°54′N, 4°34′W), Taounate (~ 347 m a.s.l.; 34°32′N, 4°34′W), and Ouazzane (~ 614 m a.s.l.; 34°48′N, 5°35′W). Seeds were sourced from a legally operating supplier and harvested at full maturity, defined by the presence of a uniform brown colour in more than 90% of seeds. The material originated from commercial cultivation; no wild sampling was carried out and no specific collection permit was required under applicable regulations.

Taxonomic identification was performed by Prof. Mohamed ADDI (Faculty of Sciences, Mohammed First University, Oujda, Morocco). A voucher specimen (HUMPOM242) was deposited in the herbarium of the Faculty of Sciences, Oujda. The study was conducted in compliance with institutional, national and international standards, including the IUCN policy statement on research involving threatened species and CITES guidelines. Seeds were cleaned by sieving and winnowing to eliminate debris and damaged units, then stored in the dark at 2–4 °C until extraction and analysis.

### Preparation of aqueous extracts

Defatted solid residues were processed to obtain aqueous extracts by decoction. Briefly, 10 g of defatted residue were combined with 40 mL of distilled water in an Erlenmeyer flask and gently boiled for 30 min. After cooling to room temperature, the mixture was filtered to remove solids. The filtrate was then dried in an oven to obtain a dry extract. Dried aqueous extracts expected to be enriched in water-soluble phenolic constituents were stored at 4 °C and protected from light prior to chemical and biological assays.

### Secondary metabolite assays

#### Total phenolic content (TPC)

Total phenolic content was determined using the Folin-Ciocalteu procedure following Elbouzidi et al. (2023), with minor adjustments. Extracts were prepared at 2 mg/mL. An aliquot of 100 µL of each extract solution was mixed with 200 µL of Folin-Ciocalteu reagent and 2.0 mL of distilled water, followed by addition of 1.0 mL of 15% (w/v) Na₂CO₃. After incubation for 2 h at room temperature in the dark, absorbance was measured at 765 nm (UV–Vis). Gallic acid served as calibration standard. Results were expressed as mg gallic acid equivalents per 100 g of dry extract (mg GAE/100 g DM).

#### Total flavonoid content (TFC)

Total flavonoids were quantified using the aluminum chloride colorimetric method. Extracts (2 mg/mL) were prepared in the appropriate solvent. Then, 500 µL of each solution were mixed with 1.5 mL methanol, 100 µL of 10% AlCl₃, and 100 µL potassium acetate (1 M); the volume was brought to 2.8 mL with distilled water. After 30 min incubation at room temperature in the dark, absorbance was recorded at 415 nm against a reagent blank (Elbouzidi et al. 2023). Quercetin was used for calibration and results were expressed as mg quercetin equivalents per g of dry extract (mg QE/g DM).

### Phytochemical profiling by HPLC–DAD

Aqueous seed extracts were profiled using high-performance liquid chromatography coupled with diode-array detection (HPLC–DAD) on an Agilent 1200 system (Agilent Technologies, Palo Alto, CA, USA) equipped with a diode-array detector. Separations were performed on a reversed-phase C18 column (4.6 mm × 250 mm, 5 µm) maintained at 30–35 °C. The mobile phase consisted of a binary system: A, ultrapure water with 0.1% formic acid, and B, acetonitrile (or methanol) containing 0.1% formic acid.

The gradient began at 95% A/5% B, progressed linearly to 10% A/90% B over 45 min, and then returned to initial conditions for re-equilibration. The flow rate was 1 mL/min. Under these conditions, highly polar compounds eluted early, moderately hydrophobic constituents (including many polyphenols) eluted between approximately 10 and 30 min, and the most hydrophobic components eluted toward the end of the program. DAD acquisition was performed within 210–280 nm, a suitable range for monitoring phenolic acids and flavonoids (Haddou et al. 2023). When enhanced separation of highly hydrophilic analytes is required, hydrophilic interaction liquid chromatography (HILIC) can be considered as a complementary approach (Loukili 2021). This chromatographic strategy enables broad identification and quantification of bioactive compounds in aqueous extracts.

### Antioxidant assays (DPPH and TAC)

Antioxidant activity of aqueous extracts was assessed using the 2,2-diphenyl-1-picrylhydrazyl (DPPH) radical scavenging assay (Haddou et al. 2024) and the phosphomolybdenum total antioxidant capacity (TAC) method (Rosam et al. 2020). Extracts were prepared in methanol at concentrations ranging from 0.032 to 2 mg/mL.

For DPPH, 0.2 mL of each dilution was mixed with 1.8 mL of DPPH solution in methanol (0.04 mg/mL), vortexed for 1 min and incubated for 20 min in the dark at room temperature. Absorbance was measured at 517 nm (Ultrospec 7000). Ascorbic acid was used as reference. Radical scavenging activity was calculated as: Inhibition (%) = [(Acontrol – Asample)/Acontrol] × 100, and IC₅₀ values were determined to compare samples (El Guerrouj et al. 2023).

For TAC, 0.3 mL of each extract dilution (0.032–2 mg/mL) was mixed with 0.6 mL of reagent solution containing 0.6 M H₂SO₄, 28 mM sodium phosphate, and 4 mM ammonium molybdate (Pellegrini et al. 2021). Tubes were incubated at 95 °C for 90 min, cooled, and absorbance was read at 695 nm. TAC was expressed as mg ascorbic acid equivalents per g of dry matter (mg AAE/g DM), and EC₅₀ values were calculated as the concentration producing 50% of the maximal response (Elbouzidi et al. 2024).

### Antibacterial and antifungal activities

Antimicrobial activity of aqueous extracts was determined using a 96-well broth microdilution assay according to Balouiri et al. (2016). Extracts were dissolved in 2% DMSO or sterile water depending on solubility, and sterilized by filtration through 0.22 µm membranes. The following bacterial strains were tested: *Staphylococcus aureus* ATCC 6538P, *Micrococcus luteus* LB 14110, *Pseudomonas aeruginosa* ATCC 15442, and *Escherichia coli* ATCC 10536. Antifungal tests were conducted against *Candida glabrata*, *Rhodotorula glutinis* ON 209167, *Aspergillus niger*, and *Geotrichum candidum.*

Twofold serial dilutions (16–0.125%) were prepared to determine MIC values. Each well received 50 µL of inoculum standardized to 105 cells/mL. Tetracycline and cycloheximide served as positive controls for bacteria and fungi, respectively. Plates were incubated at 37 °C for 24 h (bacteria) or 25 °C for 48 h (fungi). Then, 15 µL of 0.015% resazurin solution was added, followed by 2 h incubation. A change from blue to pink indicated microbial growth (Lekbach et al. 2018). All assays were performed in triplicate.

### Statistical analysis

All experiments were performed in triplicate and results are expressed as mean ± standard deviation (SD). For quantitative variables, regional differences were evaluated using one-way analysis of variance (ANOVA) followed by Tukey’s post hoc test when applicable. Statistical significance was set at *p* < 0.05.

### Molecular docking

**Fig. 1:**
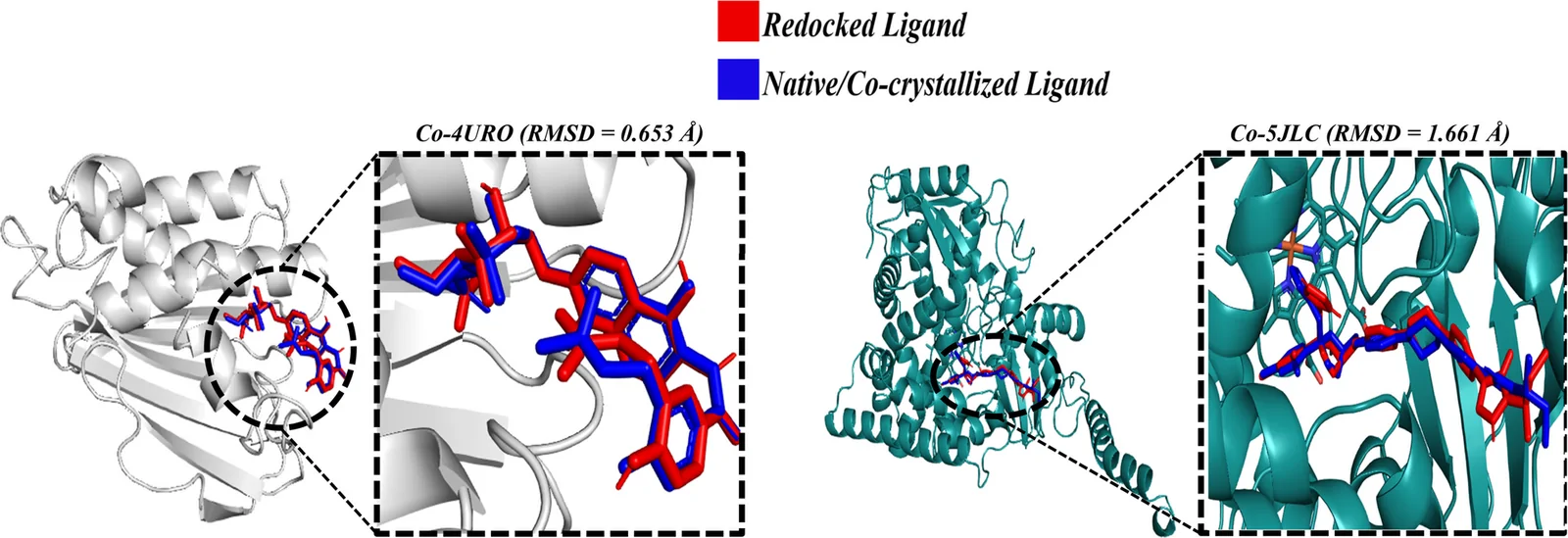
Redocking validation of the docking protocol for 4URO and 5JLC: superposition of redocked (red) and native/co-crystallized (blue) ligand poses with RMSD values of 0.653 Å (4URO) and 1.661 Å (5JLC)

Molecular docking was carried out using AutoDock Vina 1.2.0 implemented in PyRx 0.9 after standard receptor/ligand preparation, including removal of crystallographic water molecules, addition of polar hydrogens, and assignment of Kollman charges using AutoDock Tools 1.5.7 (Trott and Olson 2010; 2021); ligands were energy-minimized and converted to PDBQT prior to docking. The docking setup was validated by extracting the native/co-crystallized ligands from the two complexes used in this study (4URO and 5JLC) and redocking them into their respective binding sites with an exhaustiveness of 10, using the following grid boxes: for 4URO, center (x = −1.67, y = −0.01, z = −12.46) and size (x = 12.92, y = 17.81, z = 15.03) Å; for 5JLC, center (x = −42.35, y = 77.68, z = −23.29) and size (x = 20.44, y = 27.91, z = 11.66) Å. The redocked poses closely reproduced the experimental orientations, yielding RMSD values of 0.653 Å (4URO) and 1.661 Å (5JLC) (Fig. 1), supporting the reliability of the protocol for subsequent pose prediction and interaction analysis; 2D/3D visualization was performed with BIOVIA Discovery Studio 2024 and PyMOL 2.5.

## Results and Discussion

### Extraction yield and overall phenolic/flavonoid levels

Aqueous extraction yields differed among the three regions (Table 1). Ouazzane provided the highest yield (8.08 ± 0.24%), followed by Ketama (6.25 ± 0.19%) and Taounate (5.99 ± 0.18%). These differences are consistent with a region-associated effect on the fraction of water-extractable solids. However, because soil composition, rainfall, temperature, and agronomic practices were not directly quantified in the present study, this interpretation should be considered associative rather than causa (Ouhtit et al. 2024; Raoui et al. 2024; Haddou et al. 2023).

**Table 1:** Yield of aqueous seed extracts (%) from three Moroccan regions

Region	Yield (%)
Ketama	6.25 ± 0.19
Taounate	5.99 ± 0.18
Ouazzane	8.08 ± 0.24

The same trend was observed for global phenolic indices (Table 2). Ouazzane extracts contained the highest total phenolic content (TPC: 16.13 ± 0.48 mg GAE/100 g DM) and total flavonoid content (TFC: 6.78 ± 0.20 mg QE/100 g DM), whereas Taounate showed the lowest levels (TPC: 12.54 ± 0.38; TFC: 4.82 ± 0.14). Moroccan studies on Cannabis sativa seed extracts similarly report measurable TPC/TFC values and emphasize that extraction solvent and terroir modulate phenolic recovery (Haddou et al. 2023). In addition, detailed profiling of defatted hemp seeds confirmed that phenolics represent a relevant bioactive pool in cannabis matrices (Benkirane et al. 2023).

**Table 2:** Total phenolic content (TPC) and total flavonoid content (TFC) of aqueous seed extracts

Region	TPC (mg GAE/100 g DM)	TFC (mg QE/100 g DM)
Ketama	13.84 ± 0.42	5.24 ± 0.16
Taounate	12.54 ± 0.38	4.82 ± 0.14
Ouazzane	16.13 ± 0.48	6.78 ± 0.20

### HPLC–DAD phenolic fingerprint and regional chemotypes

HPLC–DAD profiling highlighted clear region-specific phenolic fingerprints (Table 3). Across all samples, 4-hydroxybenzoic acid was the dominant constituent (33.26–36.63%), suggesting a shared core phenolic signature for Beldia seeds. Taounate displayed a distinctive pattern marked by high vanillic acid (35.00%) and caffeic acid (20.44%), whereas Ouazzane was enriched in gallic acid (9.50%) and maintained high caffeic acid (19.50%). Ketama exhibited a broader distribution including syringic (14.00%), vanillic (14.72%), cinnamic (14.41%), and caffeic acids (8.49%) as well as catechin hydrate (4.57%).

**Table 3:** HPLC–DAD phenolic composition of aqueous seed extracts (relative %, mean ± SD)

Compound	RT (min)	Ketama (%)	Taounate (%)	Ouazzane (%)
Gallic acid	2.39 ± 0.07	4.05 ± 0.12	0.00 ± 0.00	9.50 ± 0.28
Caffeic acid	3.00 ± 0.09	8.49 ± 0.25	20.44 ± 0.61	19.50 ± 0.58
4-Hydroxybenzoic acid	3.22 ± 0.10	33.26 ± 1.00	36.63 ± 1.10	36.50 ± 1.09
Catechin hydrate	4.02 ± 0.12	4.57 ± 0.14	0.00 ± 0.00	0.00 ± 0.00
Syringic acid	4.04 ± 0.12	14.00 ± 0.42	0.00 ± 0.00	4.00 ± 0.12
Vanillic acid	5.29 ± 0.16	14.72 ± 0.44	35.00 ± 1.05	9.50 ± 0.28
Cinnamic acid	6.05 ± 0.18	14.41 ± 0.43	0.00 ± 0.00	0.00 ± 0.00
p-Coumaric acid	7.85 ± 0.24	2.89 ± 0.09	0.00 ± 0.00	5.00 ± 0.15
Ferulic acid	8.60 ± 0.26	0.00 ± 0.00	0.00 ± 0.00	6.00 ± 0.18
Sinapic acid	9.76 ± 0.29	1.44 ± 0.04	1.89 ± 0.06	4.50 ± 0.14
Quercetin-3-O-β-D-glucoside	11.26 ± 0.34	0.87 ± 0.03	2.36 ± 0.07	0.00 ± 0.00
Quercetin-3-O-glucoside	11.30 ± 0.34	0.00 ± 0.00	0.00 ± 0.00	3.00 ± 0.09
Rutin	13.09 ± 0.39	1.04 ± 0.03	0.00 ± 0.00	2.50 ± 0.07
Kaempferol	18.89 ± 0.57	0.26 ± 0.01	3.68 ± 0.11	0.00 ± 0.00

This composition aligns with literature indicating that hemp/cannabis seeds can contain diverse hydroxybenzoic and hydroxycinnamic acids together with lower-abundance flavonoid-type constituents. In the present samples, the detected flavonoids were catechin hydrate, quercetin-3-O-β-D-glucoside, quercetin-3-O-glucoside, rutin, and kaempferol, whereas phenolic acids represented the dominant fraction of the profile (Benkirane et al. 2022; 2023). Phenolic acids such as gallic and ferulic acids have documented antibacterial modes of action, including membrane perturbation and intracellular leakage, supporting their relevance for the bioactivities observed here (Borges et al. 2013). In addition, caffeic acid has been repeatedly reported to inhibit *Staphylococcus aureus* and *Escherichia coli* and to affect membrane integrity, providing a mechanistic rationale for extracts rich in this compound (Kępa et al. 2018; Andrade et al. 2015).

### Antioxidant capacity (DPPH and phosphomolybdenum TAC)

Antioxidant activity differed markedly by region (Table 4). In the DPPH assay, Ketama exhibited the strongest radical-scavenging potential (IC₅₀ = 10.25 ± 0.03 µg/mL), followed by Ouazzane (14.70 ± 0.03 µg/mL), whereas Taounate was less active (38.19 ± 0.03 µg/mL). All extracts outperformed ascorbic acid under the applied conditions (IC₅₀ = 61.43 ± 0.04 µg/mL). In contrast, TAC (reported as EC₅₀) ranked Ouazzane as the most effective reducing system (565.80 ± 5.90 mg AAE/g), consistent with its higher global phenolic indices.

**Table 4:** Antioxidant activity of aqueous seed extracts (DPPH IC₅₀ and TAC EC₅₀)

Sample	DPPH (IC₅₀, µg/mL)	TAC (EC₅₀, mg AAE/g)
Ketama	10.25 ± 0.03	494.29 ± 4.98
Taounate	38.19 ± 0.03	486.97 ± 4.97
Ouazzane	14.70 ± 0.03	565.80 ± 5.90
Ascorbic acid	61.43 ± 0.04	500.80 ± 5.73

The divergence between DPPH and TAC trends is expected because these assays probe different antioxidant mechanisms and do not respond equally to all phenolic structures. DPPH mainly reflects radical-quenching efficiency, whereas the phosphomolybdenum assay captures broader electron-transfer and reducing capacity (Prieto et al. 1999). Consequently, samples with similar total phenolic levels may rank differently depending on the relative abundance, redox potential, and structural features of individual constituents (Raoui et al. 2024; 2023). The strong DPPH activity in Ketama, despite lower TPC than Ouazzane, suggests that particular compounds such as cinnamic acid, syringic acid, and catechin hydrate may contribute disproportionately to radical scavenging.

### Antibacterial activity and MIC/MBC determination

Agar diffusion screening revealed consistent antibacterial activity for all extracts (Table 5). Ouazzane showed the largest inhibition zones against both Gram-positive and Gram-negative strains (e.g., 16.0 ± 0.2 mm against *S. aureus* and 16.8 ± 0.1 mm against *M. luteus*), while Ketama exhibited the lowest overall diameters. As commonly observed for plant phenolics, Gram-positive bacteria tended to be slightly more susceptible than Gram-negative organisms, likely due to the outer membrane permeability barrier in Gram-negative bacteria (Desbois and Smith 2010; Jia et al. 2020).

**Table 5:** Antibacterial activity (inhibition zones, mm)

Sample	*S. aureus*	*M. luteus*	*E. coli*	*P. aeruginosa*
Ketama	13.50 ± 0.40	14.50 ± 0.10	13.00 ± 0.10	13.10 ± 0.30
Taounate	14.50 ± 0.20	15.40 ± 0.10	13.60 ± 0.30	13.40 ± 0.10
Ouazzane	16.00 ± 0.20	16.80 ± 0.10	14.40 ± 0.20	14.00 ± 0.20
Negative control (DMSO)	0.00 ± 0.00	0.00 ± 0.00	0.00 ± 0.00	0.00 ± 0.00
Positive control (Gentamicin)	25.00 ± 1.20	21.00 ± 1.10	22.00 ± 0.90	25.00 ± 1.10

Broth microdilution confirmed the greater antibacterial potency of the Ouazzane extract (Table 6). It showed MIC values of 0.75 ± 0.02 mg/mL against S. aureus and 0.50 ± 0.01 mg/mL against *M. luteus*, with corresponding MBC values of 3.00 ± 0.09 and 2.00 ± 0.06 mg/mL, respectively. These results are consistent with the recognized antibacterial activity of hydroxybenzoic and hydroxycinnamic acids, including 4-hydroxybenzoic and caffeic acids (Kępa et al. 2018; Cho et al. 1998). Previous Moroccan studies on *Cannabis sativa* seed extracts also reported measurable antibacterial effects for phenolic-rich aqueous extracts (Benkirane et al. 2022; 2023).

**Table 6:** MIC and MBC of aqueous seed extracts (mg/mL)

Sample	*S. aureus*		*M. luteus*		*E. coli*		*P. aeruginosa*	
	MIC	MBC	MIC	MBC	MIC	MBC	MIC	MBC
Ketama	2.00 ± 0.06	16.00 ± 0.48	1.00 ± 0.03	8.00 ± 0.24	2.00 ± 0.06	16.00 ± 0.48	2.00 ± 0.06	16.00 ± 0.48
Taounate	1.00 ± 0.03	8.00 ± 0.24	0.50 ± 0.01	4.00 ± 0.12	2.00 ± 0.06	16.00 ± 0.48	2.00 ± 0.06	16.00 ± 0.48
Ouazzane	0.75 ± 0.02	3.00 ± 0.09	0.50 ± 0.01	2.00 ± 0.06	2.00 ± 0.06	8.00 ± 0.24	2.50 ± 0.07	8.00 ± 0.24

### Antifungal activity and MIC/MFC determination

All aqueous extracts inhibited fungal growth in agar diffusion assays (Table 7). Ouazzane exhibited the strongest overall effect, with inhibition zones of 17.4 ± 0.2 mm against *G. candidum* and 16.5 ± 0.1 mm against *A. niger.* Phenolic acids and related aromatic acids have been described as potential anti-*Candida* agents capable of affecting membrane integrity, oxidative balance, and sterol homeostasis (Guimarães and Venâncio 2022; Teodoro et al. 2015).

**Table 7:** Antifungal activity (inhibition zones, mm)

Sample	*G. candidum*	*A. niger*	*C. glabrata*	*R. glutinis*
Ketama	15.20 ± 0.40	15.00 ± 0.10	15.20 ± 0.30	14.50 ± 0.10
Taounate	17.00 ± 0.20	15.50 ± 0.10	15.70 ± 0.10	16.00 ± 0.30
Ouazzane	17.40 ± 0.20	16.50 ± 0.10	16.20 ± 0.20	15.20 ± 0.20
Negative control (DMSO)	0.00 ± 0.00	0.00 ± 0.00	0.00 ± 0.00	0.00 ± 0.00
Positive control (Cycloheximide)	20.00 ± 1.20	22.00 ± 1.00	23.00 ± 0.50	24.00 ± 1.30

MIC/MFC determinations (Table 8) showed notable potency against *G. candidum*, particularly for Taounate (MIC 0.12 ± 0.00 mg/mL; MFC 1.00 ± 0.03 mg/mL). Ketama yielded the lowest MICs against A. niger and *C. glabrata* (0.50 mg/mL), while Ouazzane generally displayed lower fungicidal endpoints for several strains. Such structure-dependent fungistatic-to-fungicidal behaviors have been reported for phenolic acids and other small aromatic phytochemicals, especially against *Candida* spp. and filamentous fungi (Guimarães and Venâncio 2022; Teodoro et al. 2015).

**Table 8:** MIC and MFC of aqueous seed extracts (mg/mL)

Sample	*G. candidum*		*A. niger*		*C. glabrata*		*R. glutinis*	
	MIC	MFC	MIC	MFC	MIC	MFC	MIC	MFC
Ketama	0.50 ± 0.01	4.00 ± 0.12	0.50 ± 0.01	4.00 ± 0.12	0.50 ± 0.01	4.00 ± 0.12	1.00 ± 0.03	8.00 ± 0.24
Taounate	0.12 ± 0.00	1.00 ± 0.03	0.50 ± 0.01	4.00 ± 0.12	0.50 ± 0.01	4.00 ± 0.12	0.25 ± 0.01	2.00 ± 0.06
Ouazzane	0.50 ± 0.01	2.00 ± 0.06	1.00 ± 0.03	3.00 ± 0.09	1.25 ± 0.04	5.00 ± 0.15	1.75 ± 0.05	5.00 ± 0.15

### Molecular docking

Molecular docking was employed to complement the in vitro antimicrobial results by providing a target-based mechanistic rationale for how the identified phenolic constituents could interact with key microbial enzymes (Khibech et al. 2025; 2025). Given the observed antibacterial and antifungal activities, two biologically relevant targets were selected to mirror these endpoints: DNA gyrase subunit B (GyrB; 4URO) to contextualize broad antibacterial effects across Gram-positive and Gram-negative strains, and lanosterol 14α-demethylase (CYP51/ERG11; 5JLC) from Candida glabrata to link antifungal activity to disruption of ergosterol biosynthesis and membrane integrity (Rosam et al. 2020).

**Fig. 2:**
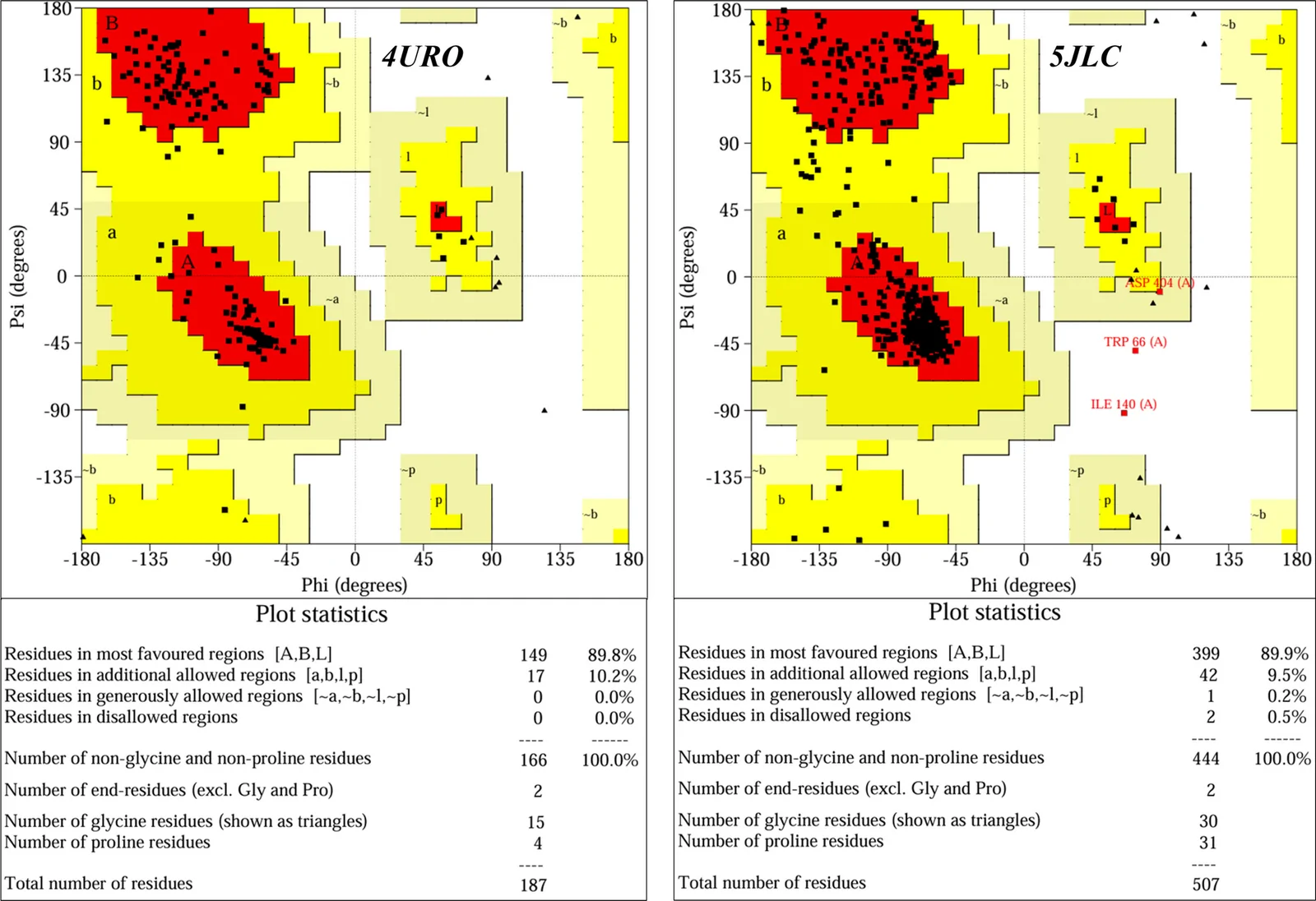
Ramachandran plot assessment of the stereochemical quality of the 4URO and 5JLC protein structures used for molecular docking

The structural quality of the two proteins used for docking is supported by the Ramachandran plot analysis (Fig. 2). For 4URO, 89.8% of residues fall within the most favoured regions and 10.2% in additionally allowed regions, with 0% in disallowed regions, indicating excellent stereochemical quality for a docking receptor. For 5JLC, 89.9% of residues are in the most favoured regions and 9.5% in additionally allowed regions, while only 0.5% fall in disallowed regions (2 residues out of 507), which remains acceptable for a large heme enzyme and suggests an overall reliable backbone geometry. Together, these results indicate that the receptor conformations are suitable for docking and that the predicted ligand protein complexes are less likely to be biased by major structural artefacts.

The binding energy results (Table 9) show a consistent trend in which flavonoid-type compounds outperform most simple phenolic acids in both targets. Among the compounds detected in the present samples, catechin hydrate, rutin, kaempferol, and quercetin glycosides (reported here under the umbrella term isoquercetin for Quercetin-3-O-β-D-glucoside/Quercetin-3-O-glucoside) were the best-scoring ligands. Against 4URO, the best scores were observed for isoquercetin (− 7.9 kcal/mol), rutin (− 7.8 kcal/mol), catechin hydrate (− 7.7 kcal/mol), and kaempferol (− 7.6 kcal/mol), while the co-crystallized ligand scored − 8.6 kcal/mol, indicating that these top ligands approach the affinity range expected for strong binders in this pocket. Against 5JLC, the same group yielded even stronger predicted affinities, led by catechin hydrate (− 9.6 kcal/mol) and rutin (− 9.6 kcal/mol), followed by isoquercetin (− 9.5 kcal/mol) and kaempferol (− 9.3 kcal/mol), while the co-crystallized ligand showed the most negative value (− 12.6 kcal/mol), as expected for a native binder. Overall, these results prioritize the flavonoid fraction as the most plausible contributor to direct target binding, whereas the abundant phenolic acids may contribute additively or indirectly to the observed antimicrobial effects.

**Table 9:** Binding energies (kcal/mol) of phenolic acids and flavonoids docked against DNA gyrase subunit B (4URO) and lanosterol 14α demethylase CYP51 (5JLC)

**Binding Energy (Kcal/mol)**		
**Molecules**	**4URO**	**5JLC**
Gallic acid	−6.1	−6.4
Caffeic acid	−7.0	−7.0
4-Hydroxybenzoic acid	−5.7	−6.5
Catechin hydrate	−7.7	−9.6
Syringic acid	−5.8	−6.3
Vanillic acid	−5.8	−6.1
Cinnamic acid	−5.8	−6.3
p-Coumaric acid	−6.3	−6.8
Ferulic acid	−6.4	−6.7
Sinapic acid	−6.3	−6.9
Isoquercetin	−7.9	−9.5
Rutin	−7.8	−9.6
Kaempferol	−7.6	−9.3
Co-4URO	−8.6	N/A
Co-5JLC	N/A	−12.6

*N/A* Not Applicable, *Isoquercetin* (Quercetin-3-O-β-D-glucoside, Quercetin-3-O-glucoside)

**Fig. 3:**
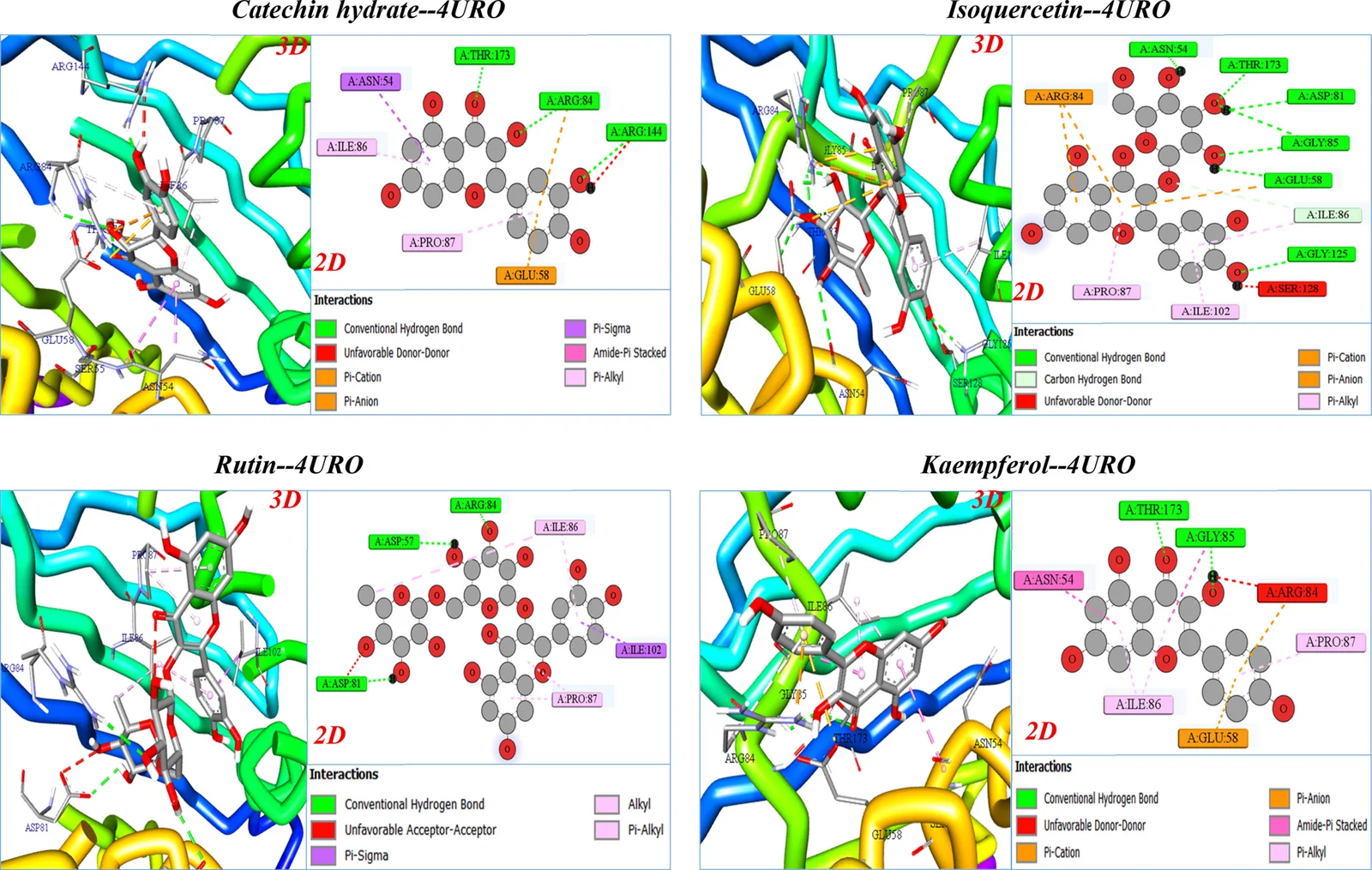
Predicted binding modes and 2D/3D interaction profiles of the top four ligands (catechin hydrate, isoquercetin, rutin, kaempferol) within the active site of 4URO

The interaction analysis for 4URO (Fig. 3) indicates that the four top scoring ligands occupy the ATPase binding region through a combination of multiple conventional hydrogen bonds and electrostatic/π type contacts with key pocket residues. Catechin hydrate is stabilized by a dense hydrogen bonding pattern involving residues such as Thr173 and positively charged residues (e.g., Arg84) along with complementary contacts to acidic/polar positions (e.g., Glu58/Asn54), which collectively supports stable anchoring within the catalytic region. Isoquercetin and rutin display broader interaction footprints, consistent with their larger, glycosylated structures, allowing them to form several hydrogen bonds with polar residues around the pocket (e.g., Asn54, Asp81, Glu58, Gly85) while simultaneously engaging hydrophobic/π interactions (including π cation contacts with Arg84) that can reinforce binding stability (Baddaoui et al. 2026; Bouammali et al. 2025). Kaempferol, despite its smaller aglycone scaffold, remains well positioned in the same site through a balanced set of hydrogen bonds and π/electrostatic contacts, suggesting that the flavonoid core itself is intrinsically compatible with the GyrB ATPase pocket. Taken together, these interaction patterns support the hypothesis that seed polyphenols could interfere with bacterial DNA replication by engaging GyrB, which is consistent with the measurable inhibition zones and MIC/MBC values obtained against both Gram positive and Gram negative bacteria.

**Fig. 4:**
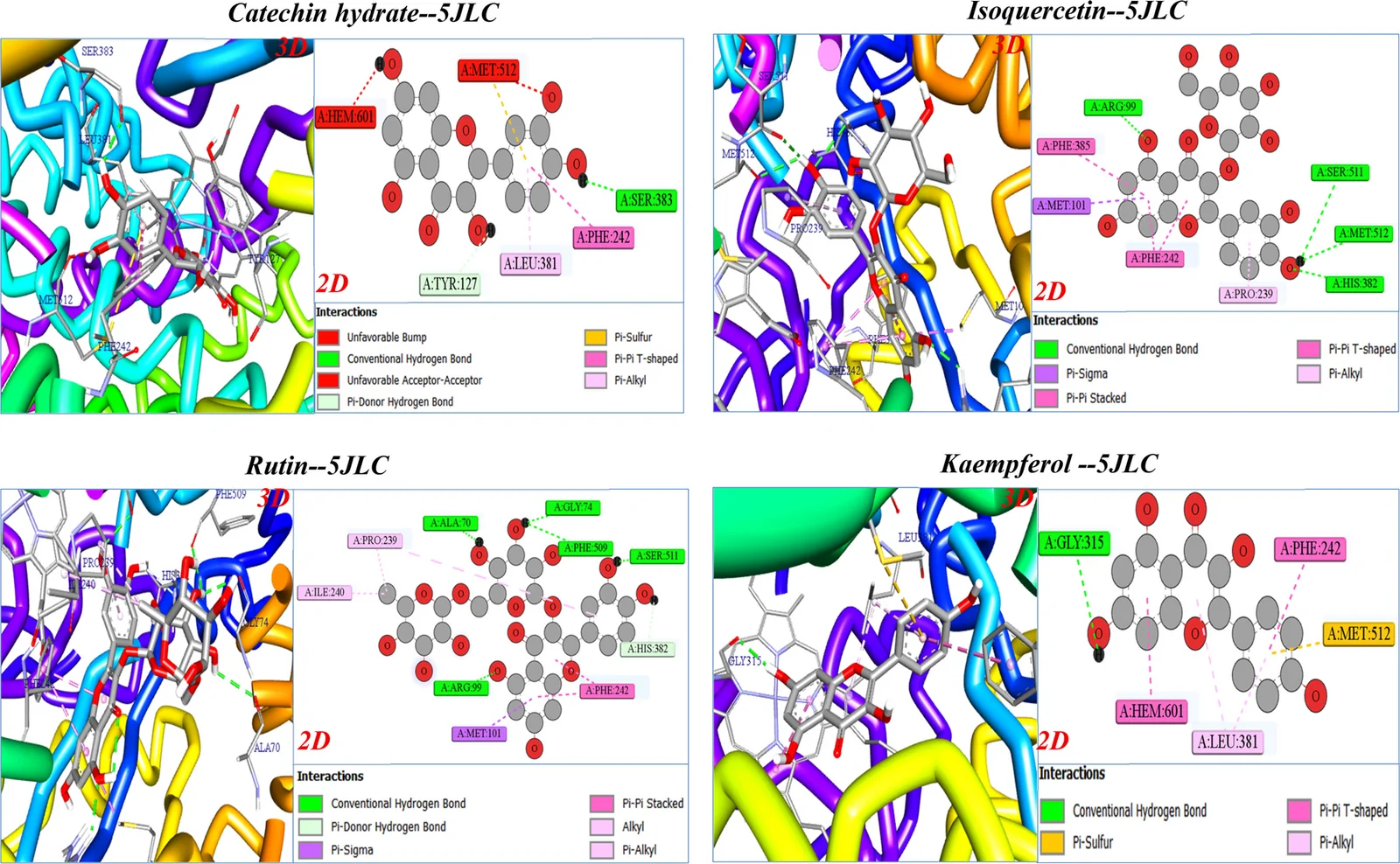
Predicted binding modes and 2D/3D interaction profiles of the top four ligands (catechin hydrate, isoquercetin, rutin, kaempferol) within the active site of 5JLC

For 5JLC (Fig. 4), the four top ligands adopt binding poses within the characteristic heme containing CYP51 cavity, where stabilization typically arises from a combination of polar anchoring and aromatic/hydrophobic packing around the substrate channel. The interaction maps show that these ligands establish multiple contacts with residues lining the active site (e.g., Ser511/Ser383, Arg99, His382, Met512, Phe242/Phe385, Leu381, Phe509) and, importantly, may approach the heme cofactor (HEM601) environment that defines the catalytic center of CYP51 (Abbaoui et al. 2024; 2025). Catechin hydrate, isoquercetin, and rutin present extensive hydrogen bonding capacity due to their multiple hydroxyl groups (and in glycosides, the sugar hydroxyls), which enables strong polar networks while the aromatic rings contribute π stacking and hydrophobic contacts with phenylalanine rich regions of the pocket; these features align with their top docking scores (≈ −9.5 to −9.6 kcal/mol).

Kaempferol shows a slightly weaker score but maintains a stable pose through key polar contacts and hydrophobic/π interactions, indicating that the flavonoid scaffold can be accommodated in the CYP51 site even without the sugar unit. Collectively, these predicted binding modes support a plausible antifungal mechanism in which polyphenols partially occupy the CYP51 substrate channel and hinder enzymatic function, which is consistent with the antifungal inhibition observed against C. glabrata in the biological assays (while recognizing that docking provides mechanistic support rather than direct proof of in vivo inhibition).

## Conclusion

This study demonstrates that aqueous extracts from defatted Beldia *(Cannabis sativa L.)* seeds exhibit pronounced region-dependent differences in extraction yield, phenolic chemotype, and bioactivity. Seeds from Ouazzane provided the highest extractability and overall TPC/TFC, Taounate displayed a chemotype enriched in caffeic and vanillic acids, and Ketama delivered the strongest DPPH radical-scavenging response. All extracts inhibited clinically relevant bacteria and fungi, with Ouazzane generally achieving larger inhibition zones and lower cidal endpoints for several strains. Docking against bacterial GyrB (4URO) and fungal CYP51 (5JLC) supported plausible contributions of catechin hydrate, quercetin glycosides, rutin, and kaempferol to the observed antimicrobial profile. The study should nonetheless be interpreted in light of its limitations: only three regions and one sampling period were examined, environmental variables were not directly measured, HPLC–DAD targeted a limited set of phenolics, and docking provides mechanistic support rather than direct biochemical proof. Overall, the data support region-guided valorization of Moroccan Beldia seed by-products and justify future studies combining broader metabolomics, controlled environmental data, and bioassay-guided fractionation.

## Data Availability

No datasets were generated or analysed during the current study.
